# Machine-Learning Approach to Optimize SMOTE Ratio in Class Imbalance Dataset for Intrusion Detection

**DOI:** 10.1155/2018/9704672

**Published:** 2018-11-01

**Authors:** Jae-Hyun Seo, Yong-Hyuk Kim

**Affiliations:** ^1^Department of Computer Science and Engineering, Wonkwang University, 460 Iksandae-ro, Iksan-si, Jeonbuk 54649, Republic of Korea; ^2^School of Software, Kwangwoon University, 20 Kwangwoon-ro, Nowon-gu, Seoul 01897, Republic of Korea

## Abstract

The KDD CUP 1999 intrusion detection dataset was introduced at the third international knowledge discovery and data mining tools competition, and it has been widely used for many studies. The attack types of KDD CUP 1999 dataset are divided into four categories: user to root (U2R), remote to local (R2L), denial of service (DoS), and Probe. We use five classes by adding the normal class. We define the U2R, R2L, and Probe classes, which are each less than 1% of the total dataset, as rare classes. In this study, we attempt to mitigate the class imbalance of the dataset. Using the synthetic minority oversampling technique (SMOTE), we attempted to optimize the SMOTE ratios for the rare classes (U2R, R2L, and Probe). After randomly generating a number of tuples of SMOTE ratios, these tuples were used to create a numerical model for optimizing the SMOTE ratios of the rare classes. The support vector regression was used to create the model. We assigned each instance in the test dataset to the model and chose the best SMOTE ratios. The experiments using machine-learning techniques were conducted using the best ratios. The results using the proposed method were significantly better than those of previous approach and other related work.

## 1. Introduction

The early IDS (intrusion detection system) [1] is divided into the host-based IDS (HIDS) and the network-based IDS (NIDS). HIDS has the advantage of analyzing the system log and resource usage information by the host and user. However, installing an IDS in each host increases the management points and wastes more resources. If network-level packet analysis is not possible and the attacker takes control of the system, the IDS may be interrupted. NIDS has advantages that it does not need to install an IDS on each host, and NIDS can perform analysis at the entire network level. However, there is a disadvantage in which it is possible to confirm only the attack via the IDS, and it is difficult to confirm the attack attempt at the system level. In early 2003, the IDS was losing the trust of users due to the problem of generating false positives. The causes of false positives are due to the development of erroneous rules, traffic irregularities, and limitations of pattern matching tests. Even though the IDS problem has not been solved to date, “pattern matching” is still being used as a basis for security solutions.

Intrusion detection attacks [2] are divided into misuse detection and anomaly detection. In misuse detection, detected attacks are compared with existing signatures in the database to determine whether they are intrusions. While misuse detection detects only the known attacks, anomaly detection detects a new type of attack that has a pattern different from the normal traffic and the known attack types.

Many researchers have studied intrusion detection. In general, researchers attempted to distinguish the normal class from attack classes using the publicly available intrusion detection evaluation dataset and to identify the exact attack type. However, the classification of rare classes in a huge real-time dataset requires a long computation time, and then it is difficult to achieve good efficiency. It is necessary to create and test many experimental datasets to improve classification performance by adjusting the class ratio.

In this paper, we present a novel method that optimally adjusts the SMOTE [3] ratios for rare classes. The number of cases for the tuple of SMOTE ratios is too large to test all the cases. For that reason, we propose the following efficient method. We randomly generated some tuples of SMOTE ratios and used these tuples to create a model using a support vector regression (SVR) [4]. We input a number of tuples for SMOTE ratios to the SVR model, and we chose the best tuple of SMOTE ratios. Experimental results using the proposed method were significantly better than those of the previous approach [5].

The contributions we make through the proposed method are given as follows. We suggest how to find the SMOTE ratios that show good performance with very few tests. Hence, we dramatically reduce the amount of computations required to find the best SMOTE ratios. We are sure that the proposed method is helpful for the study of class imbalances.

The remainder of this paper is organized as follows.  Section 2 explains the related works on the KDD CUP 1999 dataset [6] and class imbalances. In Section 3, we present the background of this research. In Section 4, we suggest a new method by creating a numerical model using sampled SMOTE ratios. In Section 5, we explain our experimental environments, procedures, and results. The paper ends with our concluding remarks in Section 6.

## 2. Related Work

### 2.1. KDD Dataset

Leung and Leckie [7] studied anomaly detection using unsupervised learning algorithms on the KDD CUP 1999 intrusion detection dataset. These researchers proposed density-based clustering and grid-based clustering algorithms. In density-based clustering, a cluster includes a minimum number of data points. The approach has the advantage of filtering outliers or finding clusters with arbitrary shapes. In the grid-based approach, all clustering operations are conducted on a grid structure. The method has the advantage of a fast computing speed. With the method, a classifier can learn from unlabeled data and detect new types of attacks that were previously unseen. The experimental results showed that the accuracy of their method is similar to one of existing methods, and the method has several advantages in terms of computational complexity.

Meng [8] studied intrusion detection machine-learning techniques on the KDD CUP 1999 dataset. There have been many studies using popular methods, such as artificial neural networks, SVM [9], and decision trees. However, these methods were rarely used in large-scale real intrusion detection systems. This researcher aimed at practical anomaly detection and conducted a comparative study with artificial neural networks, SVMs, and decision trees using the same environment as previous studies. In the analysis of the experimental results, the intrusion detection system with machine-learning techniques showed a high dependency on the test environment, and this researcher concluded that it was important to find a suitable method for applying machine-learning techniques to real environments.

Davis and Clark [10] reviewed the data preprocessing techniques used in anomaly-based network intrusion detection systems. The research focused on network traffic analysis and feature extraction/selection. Most of studies on NIDS dealt with the TCP/IP packet headers of network traffic. Time-based statistics can be derived from the headers to detect network scans, network worms, and DoS attacks. Recent, full service responses are analyzed to detect attacks targeting clients. This focuses on which attack classes can be detected by the reviewed methods. This review shows the trends that scrutinize packets to extract or select the most relevant features through targeted content parsing. These context-sensitive features are required to detect network attacks.

Staudemeyer and Omlin [11] used a long short-term memory recurrent neural network (LSTM-RNN) to evaluate the classification performance using the KDD CUP 1999 dataset. LSTM networks can learn “memory” and create a model with time series data. The LSTM is trained and tested on their modified KDD CUP 1999 dataset. The LSTM network structure and parameters were obtained through experiments. Several performance measures were used to analyze experimental results. Their results showed that LSTM-RNN can learn all the unknown attack classes in the training dataset. Furthermore, they found that both receiver operating characteristic (ROC) curves and area under the curve (AUC) were well suited for evaluating LSTM-RNN.

Kim et al. [12] proposed a system-call-language-modeling method based on LSTM for designing an anomaly-based host intrusion detection system. These researchers used an ensemble method to solve the false-alarm rates problem that was common in conventional intrusion detection systems. The method can effectively learn the semantic meaning and interactions of each system call that existing methods cannot handle. These researchers demonstrated the validity and effectiveness of their method through several tests on publicly available benchmark datasets, and their method has an advantage in that it is easy to transplant to other systems.

Kim et al. [13] investigated artificial intelligence intrusion detection systems that used the deep neural network (DNN) and conducted experiments on the KDD CUP 1999 dataset. Data preprocessing (such as data transformation and normalization was conducted) was used to input the dataset into the DNN model. When a learning model was created, the DNN was used for data refinement. The full dataset was used to verify the learning model. Performance measures, such as the accuracy, detection rate, and false-positive rate, were used to verify the detection efficiency of the DNN model, and the model showed good performance for intrusion detection.

Le et al. [14] studied deep-learning algorithms to solve the problem of machine-learning techniques (such as SVM and *k*-NN) that had high false-positive rates in intrusion detection systems. They found six optimizers that are applicable to the LSTM-RNN model to be the best suited for intrusion detection systems. The LSTM results using the Nadam optimizer were better than previous approaches, with an accuracy of 97.54%, a detection rate of 98.95%, and a false-positive rate of 9.98%. In Table 1, the studies related to intrusion detection are summarized.

Seo [5] tried to adjust the class imbalance of train data to detect attacks in the KDD 1999 intrusion dataset. He tested with machine-learning algorithms to find efficient SMOTE ratios of rare classes such as U2R, R2L, and Probe. He studied to improve the performance of classification focusing on detection of rare classes. The number of instances of rare classes in the train data was increased by 12, 9, and 1.5 times, respectively. The recall metrics of *k*-NN tests were increased to 0.11 in U2R class and 0.02 in R2L class. The metrics of SVM tests were increased to 0.02 in U2R class and 0.08 in R2L class, and those of decision tree tests were increased to 0.25.

### 2.2. Class Imbalance

In the study of Japkowicz [15], most previously designed concept-learning systems assume that a training dataset is generally well balanced. This assumption is not necessarily correct. In practice, most instances represent one class, and only a small number of instances represent other ones. These researchers tried to experimentally demonstrate that a class imbalance degrades the performance of standard classifiers. These researchers compared the performance of several methods that were previously proposed by other researchers.

Japkowicz and Stephen [16] studied class imbalance. Class imbalance has been reported to degrade the performance of some standard classifiers. They conducted a systematic study by answering the following three problems. First, they attempted to understand the concept complexity, the size of the training set, and the class imbalance level. Second, they discussed several basic resampling or cost-modifying methods to compare the efficiency of the previously proposed class imbalance problems. Finally, they conducted studies with the assumption that class imbalance problems also affected other classification systems, such as decision trees, neural networks, and SVMs.

Chawla et al. [17] studied the SMOTEBoost algorithm. In data mining, most of the datasets have the class imbalance problem, and data mining tools learn from imbalanced datasets. The classifier, which learns from a minority class with very few instances, tends to be biased towards a high accuracy in the prediction of the majority class. SMOTE is used in the design of classifiers to train unbalanced datasets. They presented a new approach to learn from imbalanced datasets by combining the SMOTE algorithm and the boosting procedure. Unlike standard boosting in which the same weight is given to all misclassified examples, SMOTEBoost generates synthetic examples from minority classes. SMOTEBoost indirectly changes the weight by updating and compensating for the skewed distribution. In the experiments with SMOTEBoost applied to several datasets with a high or moderate class imbalance, the classification performance for the minority class and the overall *F*-measure was improved.

Drummond and Holte [18] used two commonly used sampling methods for applying machine learning to imbalanced classes and misclassification costs. They adopted a performance analysis technique called cost curves to explore the interaction of oversampling and undersampling with the decision tree classifier C4.5. They showed that applying C4.5 to undersampling could establish a reasonable standard for comparing algorithms. However, it is recommended that the cheapest cost classifier becomes a part of the standard since it can be better than undersampling for relatively modest costs. Oversampling has little influence on the sensitivity and the misclassification costs have no significant effect on performance.

Zhou and Liu [19] demonstrated the effect of sampling and threshold-moving in training cost-sensitive neural networks. Both oversampling and undersampling were considered. These techniques modified the distribution of training data so that the costs of the instances were explicitly conveyed by the appearances of the instances. Threshold-moving moves the output threshold towards inexpensive classes to improve classification performance. The hard-ensemble and soft-ensemble are used for the experiments. In hard-ensembles and soft-ensembles, all classifiers vote on each class and return the class that receives the most votes. The difference between the two ensembles is that hard-ensemble uses binary votes and soft-ensemble uses real-value votes. Twenty-one UCI datasets and actual datasets were used in their experiments. The experimental results showed that as the number of classes increases, the degree of class imbalance worsens and the efficiency of classification deteriorates. Threshold-moving and the soft-ensemble showed relatively good performance in training cost-sensitive neural networks.

Liu et al. [9] used undersampling to solve the class imbalance problem. Undersampling is a very effective method to mitigate class imbalance using only a subset of the majority class. The disadvantage of the method is that instances of majority classes are ignored. They presented two algorithms to overcome the drawback. First, the EasyEnsemble algorithm samples several subsets from the majority class, trains a learner using each subset, and then combines the outputs of the learners. EasyEnsemble internally uses the AdaBoost ensemble. The BalanceCascade algorithm trains learners in sequence. At each step, instances of the majority class that are correctly classified by the current trained learners are removed from further consideration. The experimental results showed that both methods produce better solutions than the conventional class imbalance.

Burez and Van den Poel [20] attempted to solve the class imbalance problem to predict customer churn. Customer churn is caused by a customer who changes service provider. Customer churn is a highly rare event in the service industry, but it is a notably interesting and informative research area. However, the class imbalance problem in the context of data mining has not paid it considerable attention until recently. They studied how class imbalance can be better handled in churn prediction. They have conducted studies to improve the performance of random sampling and undersampling with appropriate evaluation matrices, such as AUC and lift. They compared gradient boosting, weighted random forest modeling, and some standard modeling techniques. They studied the performance of both random and advanced undersampling. They compared the specific modeling techniques of gradient boosting and weighted random forests with some standard techniques. In their experiment, the use of undersampling improved the prediction accuracy and the AUC values.

Seiffert et al. [21] had stated that class imbalance was a common problem in various applications. Several techniques had been used to mitigate class imbalance problems. They used a hybrid sampling/boosting algorithm called RUSBoost to train skewed training dataset. The algorithm was simpler and faster as an alternative of SMOTEBoost. They evaluated the performance of RUSBoost, SMOTEBoost, random undersampling, SMOTE, and AdaBoost. They chose fifteen datasets in various applications and then conducted experiments with four learners (C4.5D, C4.5N, naive Bayes (NB), and repeated incremental pruning) to produce error reduction (RIPPER) over four evaluation matrices. Both RUSBoost and SMOTEBoost were better than other methods, and RUSBoost performed equal to or better than SMOTEBoost.

Horng et al. [22] proposed an SVM-based intrusion detection system. The system combines a hierarchical clustering algorithm, a simple feature selection procedure, and an SVM technique. The clustering algorithm provided the SVM with fewer, abstracted, and higher qualified training instances. It was able to shorten the training time and improve the performance of a resultant SVM. The obtained SVM model could classify the network traffic data more accurately through the simple feature selection procedure. The KDD Cup 1999 dataset was used to evaluate the proposed system. Compared with other intrusion detection systems that are based on the same dataset, this system showed better performance in the detection of DoS and Probe attacks, and the best performance in overall accuracy. In Table 2, the studies related to class imbalance are summarized.

## 3. Background

### 3.1. KDD Dataset

The KDD CUP 1999 dataset [6] used in our experiments is a modification of data generated by the DARPA (Defense Advanced Research Projects Agency) intrusion detection evaluation program in 1988. The DARPA dataset is intercepted data that contain a wide range of attacks generated in a military network environment. The dataset has greatly contributed to the investigation and evaluation of intrusion detection. The dataset has been prepared and managed by MIT's Lincoln laboratory. In 1999, the modified DARPA dataset was used in the KDD CUP 1999 intrusion detection competition. MIT's Lincoln laboratory has a similar experimental environment to the typical U. S. Air Force LAN (local area network). Raw TCP dump data were generated over nine weeks. As in a real Air Force environment, the LAN was activated and various attacks were executed. However, there was a disadvantage in that there was no noise in the real data. However, the KDD CUP 1999 dataset served as a testbed to overcome the vulnerabilities of signature-based IDSs in detecting new attack types and attracted the attention of many researchers. The KDD CUP 1999 dataset is most widely used for the evaluation of such a system. There are many previous approaches using the dataset and it will be possible to compare the approaches with a new method.

Table 3 represents the files in the KDD CUP 1999 dataset and the details for those. The files “kddcup.data_10_percent.gz” and “corrected.gz” are used as training data and test data, respectively. The training data are compressed binary TCP dump data collected over approximately seven weeks with approximately 5 million connection records. The testing data are collected over approximately two weeks. They are composed of approximately 2 million connection records. Connection records are a collection of TCP packets flowing from the source IP to the destination IP, and these are classified into a normal or attack class. In the case of connection records belonging to an attack class, these are represented by exactly one specific attack type. The size of each connection record is approximately 100 bytes. Attack types are categorized into four classes, such as DoS, R2L, U2R, and Probe, as shown in Table 4.

### 3.2. SMOTE: Synthetic Minority Oversampling Technique

SMOTE [3] is a method of generating new instances using existing ones from rare or minority class. First, we identify the *k*-nearest neighbors in a class with a small number of instances and calculate the differences between a sample and these *k* neighbors. We multiply the differences by an arbitrary value between 0 and 1 and get a resultant value. Next, an instance that is generated using the resultant value is added to the training data. As a result, SMOTE works by adding any points that slightly move existing instances around its neighbors. In the aspect of increasing the number of instances in rare classes, SMOTE is similar to random oversampling. However, it does not regenerate the same instance. It creates a new instance by appropriately combining existing instances, thus making it possible to avoid the disadvantage of overfitting to a certain degree.

## 4. Modeling

### 4.1. Problem Definition

**Figure 1:**
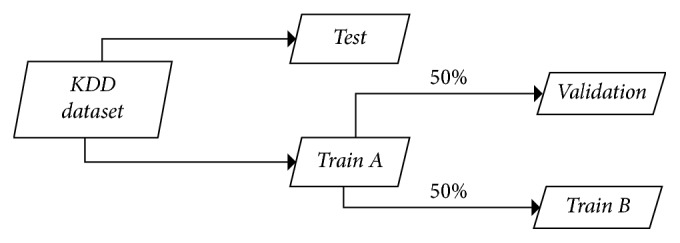
The structure of the dataset used in the proposed method.

We attempt to maximize classification performance of the KDD CUP 1999 intrusion detection dataset that has class imbalance. The dataset has severe class imbalance. Therefore, data preprocessing for adjusting the class ratio is required to alleviate the imbalance. The class imbalance can be adjusted using undersampling, oversampling, and SMOTE techniques. We use the SMOTE technique. All tuples of SMOTE ratios should be tested to optimize the ratios of each class. However, there are time and cost constraints to conduct experiments on all cases. Therefore, we try to find the tuple of SMOTE ratios that shows the best performance by experimenting with few tuples of SMOTE ratios. Formula (1) represents the method to calculate class imbalance ratio of each class.  Figure 1 shows the structure of the dataset which is used in the proposed method.  Table 5 shows class imbalance ratios of *Train A*, *Train B* which is the first half of *Train A*, *Validation* which is the second half of *Train A*, and *Test*. *Train A* is the original train data. *Train B* and *Validation* in Table 5 are basically the same. *Train B* in Table 5 shows the instances after applying the SMOTE ratios in Table 6. We define the three classes of U2R, R2L, and Probe as rare classes because the classes have relatively fewer instances than other classes.

Label cardinality of *D* is the average number of labels of the examples in *D*:

(1) $$\begin{array}{c} LC\left(D\right)=\frac{1}{\left|D\right|}\overset{\left|D\right|}{\underset{i=1}{\sum }}\left|Y_{i}\right|,\\ {\text{imbalance}\;\;\mathrm{ratio}}_{i}=\frac{\left|Y_{i}\right|}{LC\left(D\right)-\left|Y_{i}\right|}. \end{array}$$

### 4.2. Proposed Method

We attempt to optimize the SMOTE ratios of rare classes to mitigate the class imbalance. It is difficult to test all tuples of SMOTE ratios in a short period of time. Therefore, we attempt to identify an efficient method with a small number of experiments and reduce computation time.

We create an SVR model with a small number of experiments and try to get the best tuple of the SMOTE ratios by inputting enough tuples of SMOTE ratios into the model. We also verify the results through experiments. The numbers of 100 and 1,000,000, which are used in the experiments, are decided by considering computation time and 100 instances are generated randomly from a uniform distribution. We use random sampling method instead of grid one. If we can use more than 100 instances, grid sampling is not bad, but the method is not appropriate to sample very few instances uniformly. We set the ranges for the rare classes through preliminary experiments, as shown in Table 7.

We randomly generate 100 tuples of SMOTE ratios within the maximum ranges of Table 7. We conduct experiments by inputting the 100 tuples into an SVM classifier. As results, five recall values are given to each of the 100 tuples. An SVR model is created using the 100 tuples and the root mean square of the recall values. We randomly generate 1,000,000 tuples of SMOTE ratios and input them into the SVR model to derive the optimal solution. We conduct experiments to verify the quality of the best tuple.

**Figure 2:**
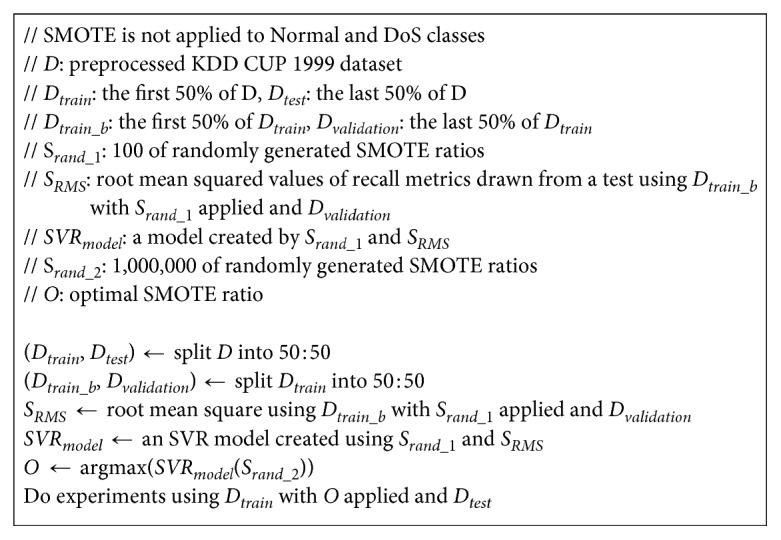
Pseudocode of the proposed method.

Formula (2) represents procedure of the proposed method. The method shows good performance with very few tests and significantly reduces the amount of computations which are required to find the best SMOTE ratios.  Figure 2 represents its pseudocode.

Procedures of the proposed methods as follows:

1. Set the ranges for the rare classes through preliminary experiments, as shown in Table 7. The ranges were searched by inputting successive 2^*t*^ where *t* is a nonnegative integer.
2. Generate randomly few tuples of SMOTE ratios from a uniform distribution (independent variable).
3. After drawing recall metrics by giving the tuples into an SVM classifier, calculate RMS with the metrics (dependent variable).
4. Create an SVR model [4] with the tuples and RMS.
5. Find the best tuple among a lot of tuples, which are generated randomly from a uniform distribution, through the SVR model.

(2) $$\begin{array}{c} \text{// }N\text{: }\mathrm{normal,}U\text{: }\mathrm{change}\;\;\mathrm{to}\;\;U2\text{R,}R\text{: }R2\text{L,}D\text{: }\mathrm{DoS,}P\text{: }\mathrm{change}\;\;\mathrm{to}\;\;\mathrm{Probe,}m\text{: }\mathrm{the}\;\;\mathrm{number}\;\;\mathrm{of}\;\;\mathrm{classes,}\\ \text{// }U_{\max}\text{,}R_{\max}\text{,}P_{\max}\text{: }\mathrm{maximum}\;\;\mathrm{range}\;\;\mathrm{of}\;\;\mathrm{each}\;\;\mathrm{class,}\\ \text{// }T_{\text{model}}\text{: }\mathrm{tuples}\;\mathrm{of}\;\;\mathrm{SMOTE}\;\;\mathrm{ratios}\;\;\mathrm{required}\;\;\mathrm{for}\;\;\mathrm{model}\;\;\mathrm{creation,}\\ \text{// }T_{\text{eval}}\text{: }\mathrm{tuples}\;\mathrm{of}\;\;\mathrm{SMOTE}\;\;\mathrm{ratios}\;\;\mathrm{required}\;\;\mathrm{to}\;\;\mathrm{evaluate}\;\;\mathrm{the}\;\;\mathrm{model,}\\ \text{// }t_{\text{best}}\text{: }\mathrm{the}\;\;\mathrm{best}\;\;\mathrm{tuple}\;\;\mathrm{among}\;\;T_{\text{eval}}\text{,}\\ U⟵\left[1,U_{\max}\right],R⟵\left[1,R_{\max}\right],P⟵\left[1,P_{\max}\right],\\ T_{\text{model}}⟵\left\{U,R,P\right\},\\ \text{RMS}=\sqrt{\frac{\sum_{i=0}^{m}{\left(\text{SVM}\_\text{Classifier}\left(T_{\text{model}}\right)\right)}_{i}^{2}}{m}},\\ \text{Model}⟵\text{SVR}\_\text{Classifier}\left(T_{\text{model}},\text{RMS}\right),\\ t_{\text{best}}⟵\arg\max\left(\text{Model}\left(T_{\text{eval}}\right)\right). \end{array}$$

**Figure 3:**
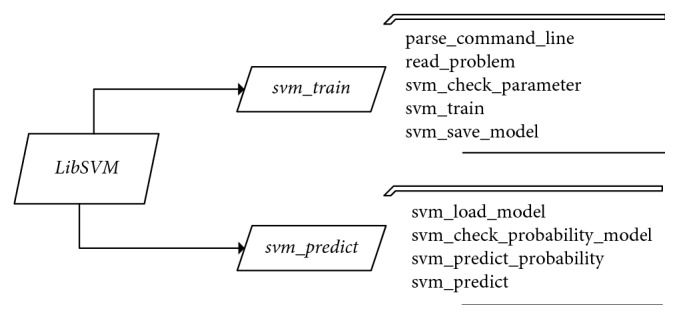
Hierarchy of the methods in LibSVM [23].

Figure 3 shows a hierarchy of the methods in LibSVM.  Table 8 represents the time complexity of SVM.  Table 9 shows the time complexity of the proposed methods.

## 5. Experiments

We randomly generate 100 tuples of SMOTE ratios and use the tuples to create an SVR model. We find the best tuple by giving 1,000,000 randomly generated tuples of SMOTE ratios into the SVR model. The experiment results with the best tuple were improved by approximately 20 percent compared with the previous approach [5]. The SVR model was generated using only 100 tuples of SMOTE ratios. As with the SVR model, the computation time was dramatically reduced and the tuple of SMOTE ratios with the highest efficiency was found.

Formula (3) gives the root mean square (RMS) using the recall values, which are the results of experiments with the 100 tuples of the SMOTE ratios of the U2R, R2L, and Probe classes. The 100 tuples are randomly generated within the range of Table 7. Variable *N* is the normal, *U* is the U2R, *R* is the R2L, *D* is the DoS, and *P* is the Probe class.  Table 10 shows parameters of SVR and SVM.  Table 11 shows parameters of RNN-LSTM.  Table 12 shows the measures drawn by creating an SVR model using the 100 tuples of SMOTE ratios and the RMS. The correlation coefficient was more than 0.7, which indicates a strong positive linear relationship. The RMSE was 0.006, which means that the difference between the expected value and the actual one is very small. Since the root relative squared error is a measure that compares the standard deviation of the actual values with the differences between the predicted and actual values, it is not a significant factor in evaluating the performance of the model.  Table 13 shows the recall metrics of experiments by the best tuple. The best tuple represents 1,000 times for the U2R, 451 times for the R2L, and 1 time for the Probe, as shown in Table 6. Table 6 shows the difference of SMOTE ratios between the proposed method and the previous one. The proposed method searches an optimal solution among a lot of SMOTE ratios, but the previous one uses only fixed SMOTE ratios.

(3) $$\begin{array}{c} \text{RMS}=\sqrt{\frac{N_{\text{recall}}^{2}+U_{\text{recall}}^{2}+R_{\text{recall}}^{2}+D_{\text{recall}}^{2}+P_{\text{recall}}^{2}}{\text{The}\;\;\mathrm{number}\;\;\mathrm{of}\;\;\mathrm{classes}}}. \end{array}$$

**Figure 4:**
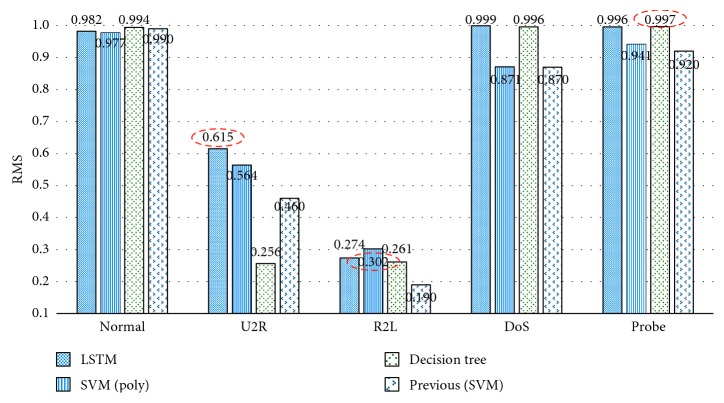
Comparison bar chart of the RNN-LSTM, SVM, decision tree, and previous SVM tests [1]. The dotted red lines represent the best recall values among rare classes.

**Figure 5:**
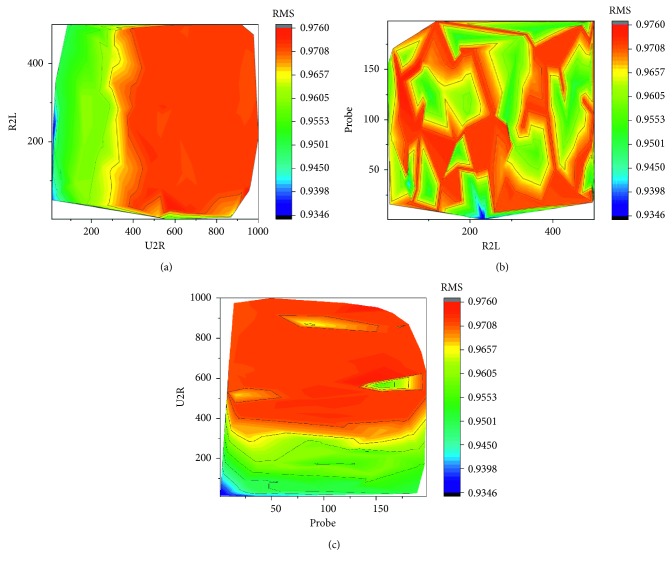
SMOTE ratios of the U2R, R2L, and Probe used to create the SVR model (RMS values). (a) U2R vs. R2L. (b) R2L vs. Probe. (c) Probe vs. U2R.

**Figure 6:**
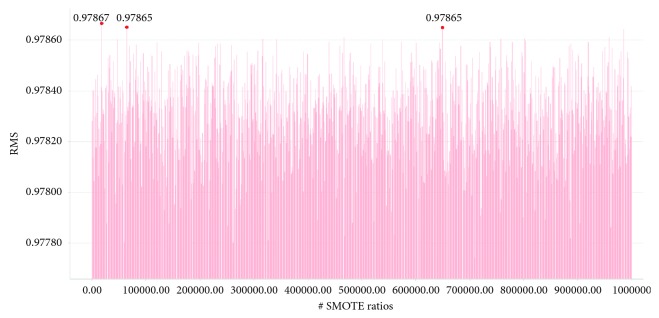
Bar chart representing RMS for 1,000,000 tuples of SMOTE ratios which are randomly generated. The red dots represent SMOTE ratios with high RMS value.

Figure 4 compares recall metrics of the proposed method with that of the previous approach [5]. RNN-LSTM is slightly superior to other methods. In the SVM tests, the performances of the U2R, R2L, and Probe classes were improved by approximately 22.6%, 58.9%, and 2.3%, respectively.  Figure 5 represents SMOTE ratios of the U2R, R2L, and Probe used to create the SVR model.  Table 14 [22] compares the proposed methods with other work by the detection rate.  Figure 6 shows a graph for the RMS of the results obtained by inputting 1,000,000 tuples of SMOTE ratios into the SVR model. The reason for defining the RMS of Formula (3) as the objective value is to make the recall values of rare classes well reflected by experimental results. An RMS of the best tuple is about 0.979.  Table 15 shows recall values of previous work [5].

Tables 16 and 17 show confusion matrix of SVM and RNN-LSTM, respectively. We conducted experiments with SVM and decision tree on the three dataset combinations of (*Train B*, *Validation*), (*Train B*, *Test*), and (*Train A*, *Test*) datasets. The results showed that SVM was better than the decision tree.  Table 16 represents recall values of the previous methods and SVM was superior to other work. Parameters and datasets of the proposed SVM test is identical to those of the previous one.

## 6. Conclusions

In this study, we have attempted to mitigate the problem of class imbalance in the KDD CUP 1999 intrusion detection dataset. As results, we obtained the best SMOTE ratios of rare classes, reduced the number of experiments by creating an SVR model, and had a significant performance improvement over the previous approach [5]. The best SMOTE ratios of rare classes drawn by the SVR model were 1,000 times for U2R, 451 times for R2L, and 1 time for Probe. The recall values for rare classes were 0.615 for the U2R in RNN-LSTM, 0.302 for the R2L in SVM, and 0.997 for the Probe in decision tree, respectively.

We proposed a new method to find the best SMOTE ratios that have high efficiency with a small number of experiments. The proposed method dramatically reduced the number of adjustments for classes. Therefore, the computation time required for the experiments could be shortened.

In future, it will be meaningful to investigate the change of test results according to the number of tuples of SMOTE ratios. We can identify better SMOTE ratios using the models created by other machine-learning techniques. Also, we will apply evolutionary computations or other metaheuristic algorithms to identify the best tuple.

## Figures and Tables

**Table 1:** Related work with KDD CUP 1999.

Authors	Year	Method
Leung and Leckie [7]	2005	Density-based and grid-based clustering
Meng [8]	2011	SVM, neural networks, and decision tree
Davis and Clark [10]	2011	Data preprocessing
Staudemeyer and Omlin [11]	2013	LSTM-RNN
Kim et al. [12]	2016	LSTM and ensemble
Kim et al. [13]	2017	DNN
Le et al. [14]	2017	DNN
Seo [5]	2017	SVM, *k*-NN, and decision tree

**Table 2:** Related work with class imbalance.

Authors	Year	Method
Japkowicz [15]	2000	Multilayer perceptron (MLP)
Japkowicz and Stephen [16]	2002	Decision tree and SVM
Chawla et al. [17]	2003	SMOTE and SMOTEBoost
Drummond and Holte [18]	2003	Decision tree
Zhou and Liu [19]	2006	Cost-sensitive neural networks
Liu et al. [9]	2009	EasyEnsemble
Burez and Van den Poel [20]	2009	Gradient boosting and random forest
Seiffert et al. [21]	2010	RUSBoost, SMOTEBoost, SMOTE, and AdaBoost
Honrng et al. [22]	2011	SVM and hierarchical clustering

**Table 3:** Train and test dataset with labels of KDD CUP 1999 dataset.

Dataset	Details
Kddcup.data.gz	Training dataset (743 MB)
Kddcup.data_10_percent.gz (23 attack types)	10% subset of training dataset (75 MB)
Corrected.gz (23 attack types)	Test dataset (45 MB)

**Table 4:** Five main categories of KDD CUP 1999 dataset.

Attacks	Descriptions
Normal	Normal traffic
DoS	Denial of service, e.g., syn flood
R2L	Unauthorized access from a remote machine, e.g., guessing password
U2R	Unauthorized access to local superuser (root) privileges, e.g., various “buffer overflow” attacks
Probing	Surveillance and other probing, e.g., port scanning

**Table 5:** Class imbalance ratios of *Train A*, *Train B*, *Validation*, and *Test* dataset.

Classes	#*Train A*	Ratio (%)	#*Train B*	Ratio (%)	#Validation	Ratio (%)	#*Test*	Ratio (%)
Normal	97,278	24.52%	48,639	10.13%	48,639	24.52%	60,593	26.15%
U2R	52	0.01%	26,026	5.17%	26	0.01%	39	0.01%
R2L	1,126	0.23%	254,476	92.70%	563	0.23%	5,993	2.09%
DoS	391,458	381.68%	195,729	58.73%	195,729	381.68%	223,298	323.61%
Probe	4,107	0.84%	4,108	0.78%	2,053	0.84%	2,377	0.82%

**Table 6:** Comparison between the proposed SMOTE ratio and the previous one.

Classes	The proposed (%)	The previous [5] (%)
Normal	—	—
U2R	100,000	12,000
R2L	49,500	900
DoS	—	—
Probe	100	150

**Table 7:** Range of SMOTE ratio for the rare classes.

	U2R	R2L	Probe
SMOTE ratio	100–100,000%	100–50,000%	100–20,000%

**Table 8:** Time complexity of the methods in LibSVM [23].

Num.	Methods		Complexity (Big-*O*)	Worst case
1	svm_train	Parse_command_line	*O*(*n*)	*O*(*n*^2^·*m*)
2		Read_problem	*O*(*mn*)	
3		SVM _check_parameter	*O*(*mn* + *n*^2^)	
4		SVM_train	*O*(*n*^2^*m*)	
5		SVM_save_model	*O*(*mn*)	

6	svm_predict	SVM_load_model	*O*(*mn*)	*O*(*n*^3^)
7		SVM_check_probability_model	*O*(1)	
8		SVM_predict_probability	*O*(*n*^2^)	
9		SVM_predict	*O*(*n*^3^)	

**Table 9:** Time complexity of the proposed methods.

Num. of the procedures of the proposed methods	Complexity (Big-*O*)	Worst case
1	// *g* is the number of experiments *O*(*g*)	*O*(*g*)
2	// *h* is the number of 100 tuples which are randomly generated. *O*(*h*)	*O*(*h*)
3	*O*(*h*) + svm_train + *O*(*h*)	*O*(*n*^2^·*h*)
4	// The time complexity of svr_train is identical to svm_train. *O*(*h*) + svr_train	*O*(*n*^2^·*h*)
5	// *k* is the number of 1,000,000 tuples which are randomly generated. // If an algorithm does not depend on *n*, which is a symbol of amounts of data, then the algorithm has constant complexity or symbolized by *O*(1) [23]. Therefore, the time complexity of svr_test is identical to *O*(1). *k*^∗^ svr_test	*O*(*k*)

**Table 10:** SVR and SVM parameters.

SVR parameters		Values	SVM parameters	Values
Batch size		100	Batch size	100
*C*		1	*c*	1
Filter type		Normalize training data	Filter type	Normalize training data
Kernel		PolyKernel	Epsilon	1.00*E*–12
RegSMOImproved optimizer	Epsilon	1.0*E*–12	Calibrator	Logistic
	Epsilon Param.	0.001	Kernel	PolyKernel
	Tolerance	0.001	Tolerance Param.	0.001

**Table 11:** RNN-LSTM parameters.

Parameters	Values
Input layers	41
Iteration (hidden layers)	82
Classes	5
Batch size	Full
Epochs	5000
Learning rate	0.001
Optimizer	RMSProp

**Table 12:** Results of the SVR model (10-fold cross-validation).

Measures		Values
Correlation coefficient		0.760
Mean absolute error		0.005
Root mean squared error		0.006
Relative absolute error		60.9%
Root relative squared error		64.6%
Standard deviation of SMOTE ratios	U2R	292.071
	R2L	158.932
	Probe	58.276

**Table 13:** Recall metrics of SVM, decision tree, and RNN-LSTM tests.

	*Train B* + *Validation*			*Train B* + *Test*			*Train A* + *Test*		
Classes	SVM	DT	LSTM	SVM	DT	LSTM	SVM	DT	LSTM
Normal	0.961	0.999	0.967	0.977	0.993	0.947	0.977	0.994	0.982
U2R	0.808	0.769	0.769	0.641	0.462	0.641	0.564	0.256	0.615
R2L	0.982	0.961	0.975	0.275	0.235	0.260	0.302	0.261	0.274
DoS	0.999	1.000	0.999	0.855	0.999	0.998	0.871	0.996	0.999
Probe	0.924	0.992	0.974	0.928	0.988	0.977	0.941	0.997	0.996

**Table 14:** Comparisons with other works by detection rate.

		Normal	U2R	R2L	DoS	Probe	Acc.	FP
These methods	SVM	97.7	56.4	30.2	87.1	94.1	88.2	2.4
	LSTM	98.2	61.5	27.4	99.9	99.6	98.1	0.4
SVM and clustering [22]		99.3	19.7	28.8	99.5	97.5	95.7	0.7
ESC-IDS [24]		98.2	14.1	31.5	99.5	84.1	95.3	1.9
KDD'99 winner [25]		99.5	13.2	8.4	97.1	83.3	91.8	0.6
KDD'99 runner-up [26]		99.4	11.8	7.3	97.5	84.5	91.5	0.6
Multiclassifier [27]		N/A	29.8	9.6	97.3	88.7	N/A	N/A
Association rule [28]		99.5	3.8	7.9	96.8	74.9	N/A	N/A

**Table 15:** Recall metrics of previous work [5].

Classes	SVM	*k*-NN	Decision tree
Normal	0.990	1.000	1.000
U2R	0.460	0.440	0.280
R2L	0.190	0.140	0.130
DoS	0.870	1.000	1.000
Probe	0.920	0.830	1.000

**Table 16:** Confusion matrix of SVM.

		Predicted class				
		Normal	U2R	R2L	DoS	Probe
Actual class	Normal	59,196	299	249	683	166
	U2R	3	22	14	0	0
	R2L	4,074	106	1,810	2	1
	DoS	28,759	0	4	194,517	18
	Probe	127	0	11	3	2,236

**Table 17:** Confusion matrix of RNN-LSTM.

		Predicted class				
		Normal	U2R	R2L	DoS	Probe
Actual class	Normal	59,528	92	657	86	230
	U2R	4	24	10	0	1
	R2L	4,316	23	1640	12	2
	DoS	20	0	2	223,169	107
	Probe	4	0	0	6	2,367

## Data Availability

The KDD CUP 1999 data used to support the findings of this study are available at http://kdd.ics.uci.edu/databases/kddcup99/kddcup99.html.
